# Correlation Between Gender of Department Chairs and Paid Parental Leave Benefits in Academic Dermatology Residency Programs

**DOI:** 10.7759/cureus.76207

**Published:** 2024-12-22

**Authors:** Karen Rofaeel, Jeffrey Ding, Marissa Joseph, Sahil Chawla, Faisal Khosa

**Affiliations:** 1 Medicine, University of British Columbia, Vancouver, CAN; 2 Dermatology, University of Toronto, Toronto, CAN; 3 Radiology, Vancouver General Hospital, Vancouver, CAN

**Keywords:** department chair, dermatology residency, gender, paid parental leave, post-graduate medical education

## Abstract

Background: Although the number of women entering dermatology residency programs is increasing, they still encounter numerous challenges and disparities, including limited career opportunities and difficulties in balancing family planning with their professional lives. Parental leave policies have been recognized for their positive impact on maternal, fetal, and familial well-being, career satisfaction, and gender equality. However, negative perceptions and a lack of awareness surrounding these policies may discourage female residents from taking parental leave during training. This study investigates the relationship between the gender of department chairs and the advertisement of paid parental leave policies in dermatology residency programs.

Methods: Data from the American Medical Association's Fellowship and Residency Electronic Interactive Database (FREIDA Online) and the Canadian Residency Match System (CaRMS) were utilized to identify accredited residency programs in the USA and Canada, respectively. Manual searches for gender of department chairs and parental leave policies in dermatology programs were performed between December 2021 and January 2022.

Results: Out of the 146 programs in the USA and 10 in Canada, most department chairs were male. Only 9.6% of programs, all from the USA, explicitly advertised paid parental leave policies specific to dermatology residents. There was a significant correlation between the gender of the department chair and the presence of specific policies, which was only observed in the northeastern region of the USA.

Conclusion: This study highlights the lack of advertised parental leave policies in dermatology residency programs and potential barriers preventing access to this crucial information. Lack of clear policies and negative perceptions may deter female residents from considering childbearing during their training or hinder them from taking parental leave when needed. Future research should explore program-specific reasons for policy advertising or omission and their perception by applicants and current residents.

## Introduction

Dermatology residency programs are seeing an increasing number of women entering the field, with significantly more female dermatology residents (64%) compared to medical school graduates (48%) noted in the last decade [1]. However, there are still many challenges women face and shortcomings of programs in supporting women's overall experiences during residency. Gender disparity has been documented in academic dermatology, research productivity, grant funding, clinical trial recruitment [2]. Outside of academia and research, women more often report being excluded for career advancement opportunities and facing difficult career decisions related to family planning [3]. Additionally, many barriers to childbearing still exist, such as negotiating parental leave, concerns on extending their length of training, negative perceptions from colleagues, and lack of additional supports like breastfeeding rooms [4,5]. Despite these challenges, many women become pregnant during residency and require parental leave. Parental leave policies have many positive impacts on fetal, maternal, and familial well-being [6]. Parental leave also impacts career satisfaction and gender equality, demonstrating the importance of advertising these policies in residency programs [3,4].

Despite the benefits of parental leave policies, negative perceptions on taking leave still affect women more than men. In a study by Sandler et al., 61% of general surgery residency program directors reported that new parenthood during residency negatively impacted a female resident’s work, increased the burden on other residents, and decreased their well-being more than their male counterparts' [7]. It is not surprising that these ideas, especially from program leadership, might influence female residents to delay childbearing until after completing their training or hinder them from requesting leave [8]. Out of 23 US dermatology programs with female leadership, 70% of these had more female faculty compared to 30% that had more male faculty. While a female leader was associated with a higher proportion of female faculty in this case, a gap in knowledge on available policies may exist, even for women in leadership positions [2]. A study by Gunn et al. found that many females in senior and representative positions in medical schools either incorrectly stated or were unaware of their institution’s parental leave policies [9]. This could be attributed to a lack of transparency from programs, difficulty finding this information online, or ambiguity in leave policies. Nonetheless, if even senior faculty members are not fully aware of such policies, this may be even more of an issue for junior students and residents who are more likely to be in childbearing ages and require leave.

Is it evident that program leadership influences both the perception of taking leave during residency and the transparency or accessibility of policies. Building on this, the goal of this study is to identify if a relationship exists between the gender of department chairs and advertisement of paid parental leave policies in their dermatology residency programs.

## Materials and methods

Data collection

All dermatology and internal medicine-dermatology residency programs accredited in the USA and Canada according to the American Medical Association’s Fellowship and Residency Electronic Interactive Database (FREIDA Online) and the Canadian Residency Match System (CaRMS), respectively, were included in this study. The programs were then manually searched between December 2021 and January 2022 to identify the gender of the department chair. If a department chair was not listed, the residency program director was used instead. Programs were excluded if there was no faculty roster, faculty profiles, or biographies, or a leadership position was not identified. Four US programs were excluded as a result of lacking this information. 

Gender was decided based on names, photos, and preferred pronouns if listed. We acknowledge that gender exists on a continuum but treated it as a binary for the purposes of this study. We searched for policies using the terms paternal, maternity/paternity, and family/childbearing absence or leave. Programs were classified as advertising paid parental leave if they provided any information on the length of leave provided. Programs that advertised policies through their institution’s general postgraduate medical education policies rather than program-specific policies were classified as “unspecific.” The US programs were stratified into four geographic regions according to the Census Regions and Divisions of the United States to assess regional differences. This was not done for Canada due to the limited number of programs available. This study was exempt from undergoing the Institutional Review Board's (IRB's) ethics approval because data is widely available online and no identifying information was used.

Statistical note

Statistical analysis was performed using SPSS version 25. Fisher’s exact test was computed for associations between gender and availability of paid parental benefits. An alpha of 0.05 was set as the cutoff for statistical significance.

## Results

A total of 146 (93.6%) programs from USA and 10 (6.4%) from Canada were included for analysis. There were 59 (37.8%) female and 97 (62.2%) male department chairs. Overall, 83 (53.2%) programs did not advertise information on paid parental benefits, 58 (37.2%) had information nonspecific for dermatology, and 15 (9.6%) had an explicitly described policy for dermatology residents.

Table 1 presents the number of programs that advertised paid parental benefits information stratified by department chairs' gender and geographic region. The numbers in brackets represent the percentage of programs that advertised, did not advertise, or had unspecific information about paid parental leave by the programs. There was a statistically significant association between gender and availability of paid parental benefit information for programs in the Northeast (p=0.047). There were no significant differences for the Midwest (p=0.219), South (p=0.683), West (p=0.812), and Canada (p=1.000). There were a greater number of male department heads in all US regions, and the opposite were observed in Canada where females outnumbered males.

**Table 1:** Gender of dermatology department chairs and number (and percentages) of programs with advertised paid parental leave benefits by regions. The Census Regions and Divisions of the United States was used to stratify programs into four regions. Midwest includes Indiana, Illinois, Michigan, Ohio, Wisconsin, Iowa, Nebraska, Kansas, North Dakota, Minnesota, South Dakota, and Missouri. Northeast includes Connecticut, Maine, Massachusetts, New Hampshire, Rhode Island, Vermont, New Jersey, New York, and Pennsylvania. South includes Delaware, District of Columbia, Florida, Georgia, Maryland, North Carolina, South Carolina, Virginia, West Virginia, Alabama, Kentucky, Mississippi, Tennessee, Arkansas, Louisiana, Oklahoma, and Texas. West includes Arizona, Colorado, Idaho, New Mexico, Montana, Utah, Nevada, Wyoming, Alaska, California, Hawaii, Oregon, and Washington. Canada includes programs in Nova Scotia, Quebec, Ontario, Manitoba, Alberta, and British Columbia.

	Gender	Not advertised, N (%)	Unspecific, N (%)	Advertised, N (%)	p
Midwest	Female	3 (18.8)	12 (75.0)	1 (6.3)	0.219
	Male	6 (28.6)	10 (47.6)	5 (23.8)	
Northeast	Female	9 (81.8)	1 (9.1)	1 (9.1)	0.047
	Male	15 (60.0)	10 (40.0)	0 (0.0)	
South	Female	8 (47.1)	7 (41.2)	2 (11.8)	0.683
	Male	19 (57.6)	11 (33.3)	3 (9.1)	
West	Female	6 (75.0)	1 (12.5)	1 (12.5)	0.812
	Male	8 (57.1)	4 (28.6)	2 (14.3)	
Canada	Female	5 (83.3)	1 (16.7)	0 (0.0)	1.000
	Male	3 (75.0)	1 (25.0)	0 (0.0)	

## Discussion

This study aimed to find a relationship between the gender of department chairs of dermatology residency programs in the USA and Canada and the advertising of paid parental leave by the programs. Consistent with previous trends, we found that males represented majority of leadership positions in dermatology departments and postgraduate medical education. Importantly, only a minority (9.6%) of programs, none of which were Canadian, advertised paid parental leave policies specific to dermatology programs. Only programs in the Northeast had a significant correlation between the gender of the department chair and advertisement of specific policies. While we did not find a correlation between gender of the department chair and availability of program policies in any other US regions or in Canada, our results showed that advertising these policies was not the norm by majority of programs.

In contrast to our own findings, a similar study by Hui et al. did find a significant correlation between the gender of department chairs and advertising paid parental leave in radiology residency programs, where female department chairs were more likely (69%) to advertise benefits compared to male counterparts (38%) [10]. This suggests that increasing the number of females in leadership positions may also lead to increased advertisement of benefits, which would work towards decreasing gender differences in multiple ways. Their results also highlight that there are clear differences between different specialities when it comes to paid parental leave [11-13].

Since we could not readily find paid parental leave policies online, this could indicate that programs are either not publicly sharing this information or potential barriers for residents to access this might exist. There are numerous positive impacts of offering parental leave and making these policies widely known to residents. For example, Baker et al. found a positive correlation between parental leave policies and career satisfaction for women completing Mohs Micrographic Surgery (MMS) fellowships. Females were also less likely to report being “very satisfied” with their careers compared to their male counterparts due to a variety of reasons, such as implicit gender bias, patients’ perception of female doctors, and not being recognized for their skills. These results suggest that optimizing parental leave policies and continuing to address systemic gender bias will positively impact women’s career satisfaction [3]. Although this study focused on female MMS fellows, these results can also be applied to female dermatology residents as these policies could establish early career satisfaction and encourage residents to pursue fellowship positions or further career advancement opportunities in the future. This is especially important because female retention and work satisfaction may contribute to decreasing the gender pay gap and perceived gender inequalities [14].

A limitation of our study was the reliance on photos and names available online to identify gender as a binary, which does not capture the complex gender experiences these individuals may have. Additionally, our study only focused on whether programs advertised their policies and did not assess more nuanced details, such as the length of leave, using sick leave, vacation days, or unpaid leave, and financial compensation. The length of leave allowed seems particularly important as Stack et al. found that female residents who took more than eight weeks of leave were less likely to have negative consequences afterwards, such as burnout and postpartum depression, and were able to breastfeed for longer. Furthermore, they were more likely to be satisfied with their decision to become parents in residency and felt supported by their colleagues and program leadership [8]. While the benefits of parental leave are clear, there are still evident financial and professional barriers limiting women. Clear program policies are needed to address how work will be redistributed among residents and fellows. 

## Conclusions

In conclusion, promoting transparency about paid parental leave policies is crucial for fostering inclusive and supportive residency environments. Future studies may survey why programs might choose to advertise or omit these policies and how this is perceived by applicants and current residents. This would encourage programs to take meaningful steps to reduce barriers to childbearing by normalizing leave during residency, creating a more supportive workplace, and fostering healthier work-life balance.
